# Monotonicity of the number of positive entries in nonnegative matrix powers

**DOI:** 10.1186/s13660-018-1833-5

**Published:** 2018-09-21

**Authors:** Qimiao Xie

**Affiliations:** College of Ocean Science and Engineering, Shanghai Maritime University, Shanghai, China

**Keywords:** 15B36, 15B48, Nonnegative matrix, Power, Monotonicity

## Abstract

Let *A* be a nonnegative matrix of order *n* and $f(A)$ denote the number of positive entries in *A*. We prove that if $f(A)\leq3$ or $f(A)\geq n^{2}-2n+2$, then the sequence $\{f(A^{k})\}_{k=1}^{\infty}$ is monotonic for positive integers *k*.

## Introduction

A matrix is *nonnegative (positive)* if all of its entries are nonnegative (positive) real numbers. Nonnegative matrices have many attractive properties and are important in a variety of applications [1, 2]. For two nonnegative matrices *A* and *B* of the same size, the notation $A\geq B$ or $B\leq A$ means that $A-B$ is nonnegative.

A *sign pattern* is a matrix whose entries are from the set $\{ +, -, 0\}$. In a talk at the 12th ILAS conference (Regina, Canada, June 26–29, 2005), Professor Xingzhi Zhan posed the following problem.

### Problem

([4], p. 233)

*Characterize those sign patterns of square nonnegative matrices* *A* *such that the sequence* $\{f(A^{k})\}_{k=1}^{\infty}$ *is nondecreasing*.

A nonnegative square matrix *A* is said to be *primitive* if there exists a positive integer *k* such that $A^{k}$ is positive. If we denote by $f(A)$ the number of positive entries in *A*, it seems that the sequence $\{f(A^{k})\}_{k=1}^{\infty}$ is increasing for any primitive matrix *A*. However, Šidák [3] observed that there is a primitive matrix *A* of order 9 satisfying $f(A)=18>f(A^{2})=16$. This is the motivation for us to investigate the nonnegative matrix *A* such that $\{f(A^{k})\}_{k=1}^{\infty}$ is monotonic. It is reasonable to expect that the sequence will be monotonic when $f(A)$ is too small or too large.

Since the value of each positive entry in *A* does not affect $f(A^{k})$ for all positive integers *k*, it suffices to consider the 0–1 matrix, i.e., the matrix whose entries are either 0 or 1. Denote by $E_{ij}$ the matrix with its entry in the *i*th row and *j*th column being 1 and with all other entries being 0. For simplicity we use 0 to denote the zero matrix whose size will be clear from the context.

## Main results

Let *A* be a nonnegative square matrix. We will use the fact that if $A^{2}\geq A\ (A^{2}\leq A)$, then $A^{k+1}\geq A^{k}\ (A^{k+1}\leq A^{k})$ for all positive integers *k* and thus $\{f(A^{k})\}_{k=1}^{\infty}$ is increasing (decreasing).

### Theorem 1

*Let* *A* *be a* 0*–*1 *matrix of order* *n*. *If* $f(A)\leq2$, *then the sequence* $\{f(A^{k})\}_{k=1}^{\infty}$ *is decreasing*.

### Proof

The case $f(A)=0$ is trivial.

If $f(A)=1$, then $A=E_{ij}$, $1\leq i,j\leq n$. Thus, for $k=2,3,\ldots$ ,

$$A^{k}=E_{ij}^{k}= \textstyle\begin{cases} E_{ii},& i=j;\\ 0,& i\neq j, \end{cases} $$

which implies that $\{f(A^{k})\}_{k=1}^{\infty}$ is decreasing. Next suppose $f(A)=2$.

Since $\{f(A^{k})\}_{k=1}^{\infty}$ is invariant under permutation similarity or transpose of *A*, it suffices to consider the following cases.

(1) $A=E_{11}+E_{22}$. Then $A^{2}=A$.

(2) $A=E_{11}+E_{12}$. Then $A^{2}=A$.

(3) $A=E_{11}+E_{23}$. Then $A^{2}=E_{11}\leq A$.

(4) $A=E_{12}+E_{13}$. Then $A^{2}=0$.

(5) $A=E_{12}+E_{21}$. Then $A^{k}=E_{11}+E_{22}$ for all even *k*, $A^{k}=A$ for all odd *k*.

(6) $A=E_{12}+E_{23}$. Then $A^{2}=E_{13}$, $A^{3}=0$.

(7) $A=E_{12}+E_{34}$. Then $A^{2}=0$.

It can be seen that in each case $\{f(A^{k})\}_{k=1}^{\infty}$ is decreasing. This completes the proof. □

### Theorem 2

*Let* *A* *be a* 0*–*1 *matrix of order* *n*. *If* $f(A)=3$, *then the sequence* $\{f(A^{k})\}_{k=1}^{\infty}$ *is monotonic*.

### Proof

Under permutation similarity and transpose, it suffices to consider the following cases.

(1) $A=E_{11}+E_{22}+E_{33}$. Then $A^{2}=A$.

(2) $A=E_{11}+E_{22}+E_{12}$. Then $A^{2}=A+E_{12}\geq A$.

(3) $A=E_{11}+E_{22}+E_{13}$. Then $A^{2}=A$.

(4) $A=E_{11}+E_{22}+E_{34}$. Then $A^{2}=E_{11}+E_{22}\leq A$.

(5) $A=E_{11}+E_{12}+E_{13}$. Then $A^{2}=A$.

(6) $A=E_{11}+E_{12}+E_{21}$. Then $A^{2}=A+E_{11}+E_{22}\geq A$.

(7) $A=E_{11}+E_{12}+E_{31}$. Then $A^{2}=A+E_{32}\geq A$.

(8) $A=E_{11}+E_{12}+E_{23}$. Then $A^{k}=E_{11}+E_{12}+E_{13}$ for all $k\geq2$.

(9) $A=E_{11}+E_{12}+E_{32}$. Then $A^{2}=E_{11}+E_{12}\leq A$.

(10) $A=E_{11}+E_{12}+E_{34}$. Then $A^{2}=E_{11}+E_{12}\leq A$.

(11) $A=E_{11}+E_{23}+E_{24}$. Then $A^{2}=E_{11}\leq A$.

(12) $A=E_{11}+E_{23}+E_{32}$. Then $A^{k}=E_{11}+E_{22}+E_{33}$ for all even *k*, $A^{k}=A$ for all odd *k*.

(13) $A=E_{11}+E_{23}+E_{34}$. Then $A^{2}=E_{11}+E_{24}$, $A^{k}=E_{11}$ for all $k\geq3$.

(14) $A=E_{11}+E_{23}+E_{45}$. Then $A^{2}=E_{11}\leq A$.

(15) $A=E_{12}+E_{13}+E_{14}$. Then $A^{2}=0$.

(16) $A=E_{12}+E_{13}+E_{21}$. Then $A^{k}=E_{11}+E_{22}+E_{23}$ for all even *k*, $A^{k}=A$ for all odd *k*.

(17) $A=E_{12}+E_{13}+E_{41}$. Then $A^{2}=E_{42}+E_{43}$, $A^{3}=0$.

(18) $A=E_{12}+E_{13}+E_{23}$. Then $A^{2}=E_{13}\leq A$.

(19) $A=E_{12}+E_{13}+E_{24}$. Then $A^{2}=E_{14}$, $A^{3}=0$.

(20) $A=E_{12}+E_{13}+E_{42}$. Then $A^{2}=0$.

(21) $A=E_{12}+E_{13}+E_{45}$. Then $A^{2}=0$.

(22) $A=E_{12}+E_{21}+E_{34}$. Then $A^{k}=E_{11}+E_{22}$ for all even *k*, $A^{k}=E_{12}+E_{21}$ for all odd $k\geq3$.

(23) $A=E_{12}+E_{23}+E_{31}$. Then

$$A^{k}= \textstyle\begin{cases} E_{11}+E_{22}+E_{33},& k\equiv0\ (\mathrm{mod} 3);\\ A, & k\equiv1\ (\mathrm{mod} 3);\\ E_{13}+E_{21}+E_{32},& k\equiv2\ (\mathrm{mod} 3). \end{cases} $$

(24) $A=E_{12}+E_{23}+E_{34}$. Then $A^{2}=E_{13}+E_{24}$, $A^{3}=E_{14}$, $A^{4}=0$.

(25) $A=E_{12}+E_{23}+E_{45}$. Then $A^{2}=E_{13}$, $A^{3}=0$.

(26) $A=E_{12}+E_{34}+E_{56}$. Then $A^{2}=0$.

Since in each case $\{f(A^{k})\}_{k=1}^{\infty}$ is either increasing or decreasing, this completes the proof. □

### Corollary 3

*Let* *A* *be a* 0*–*1 *matrix of order* 2. *Then the sequence* $\{f(A^{k})\}_{k=1}^{\infty}$ *is monotonic*.

### Remark

When *A* is of order $n\geq3$ with $f(A)=4$, the following example shows that $\{f(A^{k})\}_{k=1}^{\infty}$ may not be monotonic. Consider

$$A=E_{12}+E_{13}+E_{21}+E_{31}. $$

Direct computation shows that

$$A^{2}=2E_{11}+E_{22}+E_{23}+E_{32}+E_{33},\qquad A^{3}=2A. $$

Thus $f(A)=4< f(A^{2})=5>f(A^{3})=4$.

On the one hand, Theorems 1 and 2 show that $\{f(A^{k})\}_{k=1}^{\infty}$ is monotonic when $f(A)\leq3$. On the other hand, $\{f(A^{k})\}_{k=1}^{\infty}$ is expected to be also monotonic when $f(A)$ is large enough. Next we discuss the number of positive entries that *A* has to guarantee the sequence increasing.

The *permanent* of a matrix $A=(a_{ij})_{n\times n}$ is defined as

$$\operatorname {per}A=\sum_{\sigma\in S_{n}}\prod_{i=1}^{n}a_{i,\sigma(i)}, $$

where $S_{n}$ is the set of permutations of the integers $1,2,\ldots,n$. First we have the following important fact.

### Lemma 4

*Let* *A* *be a* 0*–*1 *matrix of order* *n*. *If* $\operatorname {per}A>0$, *then the sequence* $\{f(A^{k})\}_{k=1}^{\infty}$ *is increasing*.

### Proof

Since *A* is a 0–1 matrix with $\operatorname {per}A>0$, there exists a permutation matrix *P* such that $A\geq P$. Now let $A=P+B$, where *B* is also a 0–1 matrix. Then $A^{k+1}=A\cdot A^{k}=(P+B)A^{k}=P\cdot A^{k}+B\cdot A^{k}\geq P\cdot A^{k}$ for all positive integers *k*. Thus $f(A^{k+1})\geq f(P\cdot A^{k})=f(A^{k})$, which implies that $\{ f(A^{k})\}_{k=1}^{\infty}$ is increasing. □

### Theorem 5

*Let* *A* *be a* 0*–*1 *matrix of order* *n*. *If* $f(A)\geq n^{2}-2n+2$, *then the sequence* $\{f(A^{k})\}_{k=1}^{\infty}$ *is increasing*.

### Proof

First if $\operatorname {per}A>0$, by Lemma 4, $\{f(A^{k})\} _{k=1}^{\infty}$ is increasing.

Next suppose $\operatorname {per}A=0$. Then by the Frobenius–König theorem [4, p. 46], *A* has an $r\times s$ zero submatrix with $r+s=n+1$. Since $f(A)\geq n^{2}-2n+2$, *A* has at most $2n-2$ zero entries. Thus $rs\leq2n-2$. It can be seen that *r* and *s* must be one of the following solutions.

(1) $r=1$, $s=n$;

(2) $r=n$, $s=1$;

(3) $r=2$, $s=n-1$;

(4) $r=n-1$, $s=2$.

If $r=1$, $s=n$ or $r=n$, $s=1$, i.e., *A* has a zero row or a zero column, then *A* is permutation similar to a matrix of the form

$$\left[\begin{array}{cc} B & C\\ 0 & 0 \end{array}\right]$$

or its transpose, where *B* is of order $n-1$ and *C* is a column vector. Since *A* has at most $2n-2$ zero entries, *B* has at most $n-2$ zero entries. Then there exists a permutation matrix *Q* of order $n-1$ such that $B\geq Q$. Note that

$$\begin{array}{rl} {\left[\begin{array}{cc} B & C\\ 0 & 0 \end{array}\right]}^{k+1} & =\left[\begin{array}{cc} B & C\\ 0 & 0 \end{array}\right]{\left[\begin{array}{cc} B & C\\ 0 & 0 \end{array}\right]}^{k}=\left[\begin{array}{cc} B & C\\ 0 & 0 \end{array}\right]\left[\begin{array}{cc} B^{k} & B^{k-1}C\\ 0 & 0 \end{array}\right]\\ & \geq \left[\begin{array}{cc} Q & C\\ 0 & 0 \end{array}\right]\left[\begin{array}{cc} B^{k} & B^{k-1}C\\ 0 & 0 \end{array}\right]=\left[\begin{array}{cc} QB^{k} & QB^{k-1}C\\ 0 & 0 \end{array}\right]. \end{array}$$

Thus

$$\begin{array}{rl} f\left(A^{k+1}\right) & =f\left({\left[\begin{array}{cc} B & C\\ 0 & 0 \end{array}\right]}^{k+1}\right)\geq f\left(\left[\begin{array}{cc} QB^{k} & QB^{k-1}C\\ 0 & 0 \end{array}\right]\right)\\ & =f\left(QB^{k}\right)+f\left(QB^{k-1}C\right)=f\left(B^{k}\right)+f\left(B^{k-1}C\right)\\ & =f\left(\left[\begin{array}{cc} B^{k} & B^{k-1}C\\ 0 & 0 \end{array}\right]\right)=f\left({\left[\begin{array}{cc} B & C\\ 0 & 0 \end{array}\right]}^{k}\right)=f\left(A^{k}\right) \end{array}$$

for all positive integers *k*, which implies that $\{f(A^{k})\} _{k=1}^{\infty}$ is increasing.

If $r=2$, $s=n-1$ or $r=n-1$, $s=2$, then *A* is permutation similar to one of the matrices $A_{1}, A_{2}, A_{1}^{T}, A_{2}^{T}$, where

$$A_{1}=\left[\begin{array}{cccc} 0 & \cdots & 0 & 1\\ 0 & \cdots & 0 & 1\\ 1 & \cdots & 1 & 1\\ \vdots & \ddots & \vdots & \vdots \\ 1 & \cdots & 1 & 1 \end{array}\right],\;A_{2}=\left[\begin{array}{cccc} 1 & 0 & \cdots & 0\\ 1 & 0 & \cdots & 0\\ 1 & 1 & \cdots & 1\\ \vdots & \vdots & \ddots & \vdots \\ 1 & 1 & \cdots & 1 \end{array}\right].$$

Direct computation shows that $A_{1}^{2}\geq A_{1}, A_{2}^{2}\geq A_{2}$. Thus $\{ f(A^{k})\}_{k=1}^{\infty}$ is increasing. This completes the proof. □

### Remark

When $f(A)=n^{2}-2n+1$, the following example shows that $\{f(A^{k})\}_{k=1}^{\infty}$ may not be increasing. Consider

$$A=\left[\begin{array}{cccc} 0 & 0 & \cdots & 0\\ 1 & 0 & \cdots & 0\\ 1 & 1 & \cdots & 1\\ \vdots & \vdots & \ddots & \vdots \\ 1 & 1 & \cdots & 1 \end{array}\right].$$

Direct computation shows that $f(A)=n^{2}-2n+1>f(A^{2})=n^{2}-2n$.

## Conclusion

This paper considers the number of positive entries $f(A)$ in a nonnegative matrix *A* and deals with the question of whether the sequence $\{f(A^{k})\} _{k=1}^{\infty}$ is monotonic. We prove that if $f(A)\leq3$ or $f(A)\geq n^{2}-2n+2$, then the sequence must be monotonic. Some examples show that if $4\leq f(A)\leq n^{2}-2n+1$ when $n\geq3$, then the sequence may not be monotonic.
